# Microfluidic Diffusional Sizing (MDS) Measurements of Secretory Neutralizing Antibody Affinity Against SARS-CoV-2

**DOI:** 10.1007/s10439-024-03478-0

**Published:** 2024-03-08

**Authors:** Cara O’Mahoney, Ian Watt, Sebastian Fiedler, Sean Devenish, Sujata Srikanth, Erica Justice, Tristan Dover, Delphine Dean, Congyue Peng

**Affiliations:** 1 Department of Bioengineering, Clemson University, Clemson, SC USA; 2 Fluidic Analytics, Cambridge, UK; 3 Center for Innovative Medical Devices and Sensors, Clemson University, Clemson, SC USA

**Keywords:** SARS-CoV-2, Neutralizing antibody, Microfluidic diffusional sizing, Saliva

## Abstract

SARS-CoV-2 has rampantly spread around the globe and continues to cause unprecedented loss through ongoing waves of (re)infection. Increasing our understanding of the protection against infection with SARS-CoV-2 is critical to ending the pandemic. Serological assays have been widely used to assess immune responses, but secretory antibodies, the essential first line of defense, have been studied to only a limited extent. Of particular interest and importance are neutralizing antibodies, which block the binding of the spike protein of SARS-CoV-2 to the human receptor angiotensin-converting enzyme-2 (ACE2) and thus are essential for immune defense. Here, we employed Microfluidic Diffusional Sizing (MDS), an immobilization-free technology, to characterize neutralizing antibody affinity to SARS-CoV-2 spike receptor-binding domain (RBD) and spike trimer in saliva. Affinity measurement was obtained through a contrived sample and buffer using recombinant SARS-CoV-2 RBD and monoclonal antibody. Limited saliva samples demonstrated that MDS applies to saliva neutralizing antibody measurement. The ability to disrupt a complex of ACE2-Fc and spike trimer is shown. Using a quantitative assay on the patient sample, we determined the affinity and binding site concentration of the neutralizing antibodies.

**Supplementary Information:**

The online version contains supplementary material available at 10.1007/s10439-024-03478-0.

## Introduction

Severe acute respiratory syndrome coronavirus 2 (SARS-CoV-2) has spread quickly and cost the lives of more than 6 million around the globe (World Health Organization, 2023). A cohort study of 509 patients revealed that SARS-CoV-2 infection elicits immune responses; IgG, IgM, and IgA antibodies were detected after symptom onset [42]. Serum IgG against SARS-CoV-2 spike receptor-binding domain (RBD) is positively associated with patient survival. Serum IgA against spike protein trimer significantly reduces the time needed to clear the virus in the infected patients [42].

Similarly, mucosal immune response, total IgA, and neutralizing antibody are detectable after four-day post-diagnosis [7]. Secretory neutralizing antibodies disrupt the binding of the spike protein to ACE2, thus preventing the virus from entering the cells. They are the critical first defense for respiratory viruses [40]. In patients with SARS-CoV-2 infection, higher mucosal neutralizing antibody corresponds to a lower virus load [7]. The secreted neutralizing antibodies are absent in the bloodstream and, therefore, cannot be detected from the serology test [6]. Assessment of neutralizing antibodies in the mucosal surface will provide critical insights in addition to the serological evaluation and may spark novel mucosal antibody-based therapeutic intervention [26, 46]. Nasal swabs have been used to assess secretory antibodies against SARS-CoV-2. However, saliva can be an alternative as it does not require invasive collection techniques from trained personnel. Donors are less resistant to saliva collection, and it has been demonstrated to be a reliable source to provide the IgG and IgA titer against SARS-CoV-2 [21].

To reliably predict the protection through neutralizing antibodies, assays have been developed. They can be categorized into (1) measuring the inhibition of ACE2 and spike RBD/S1 complex or (2) the inhibition of virus entry to the host cells, virus propagation, and plaque count. Head-to-head comparisons of these methods have been done by Khoury and colleagues and Perkmann and colleagues [24, 35]. Neutralization tests using the live SARS-CoV-2 virus require a high level of biosafety regulation. Currently, surrogate viruses or pseudo-viral particles have been an alternative to the active virus for neutralization tests [9, 30, 44]. These tests circumvent active SARS-CoV-2 viruses and provide a qualitative estimation by the percentage of signal reduction compared to the positive controls. However, these immunoassays are achieved through multiple steps and sometimes involve cell culture [9]. Furthermore, the surrogate virus neutralization test based on surface adsorption has limitations in characterizing the binding affinity and the number of binding sites distinctive to the mucosal antibodies [40].

**Fig. 1:**
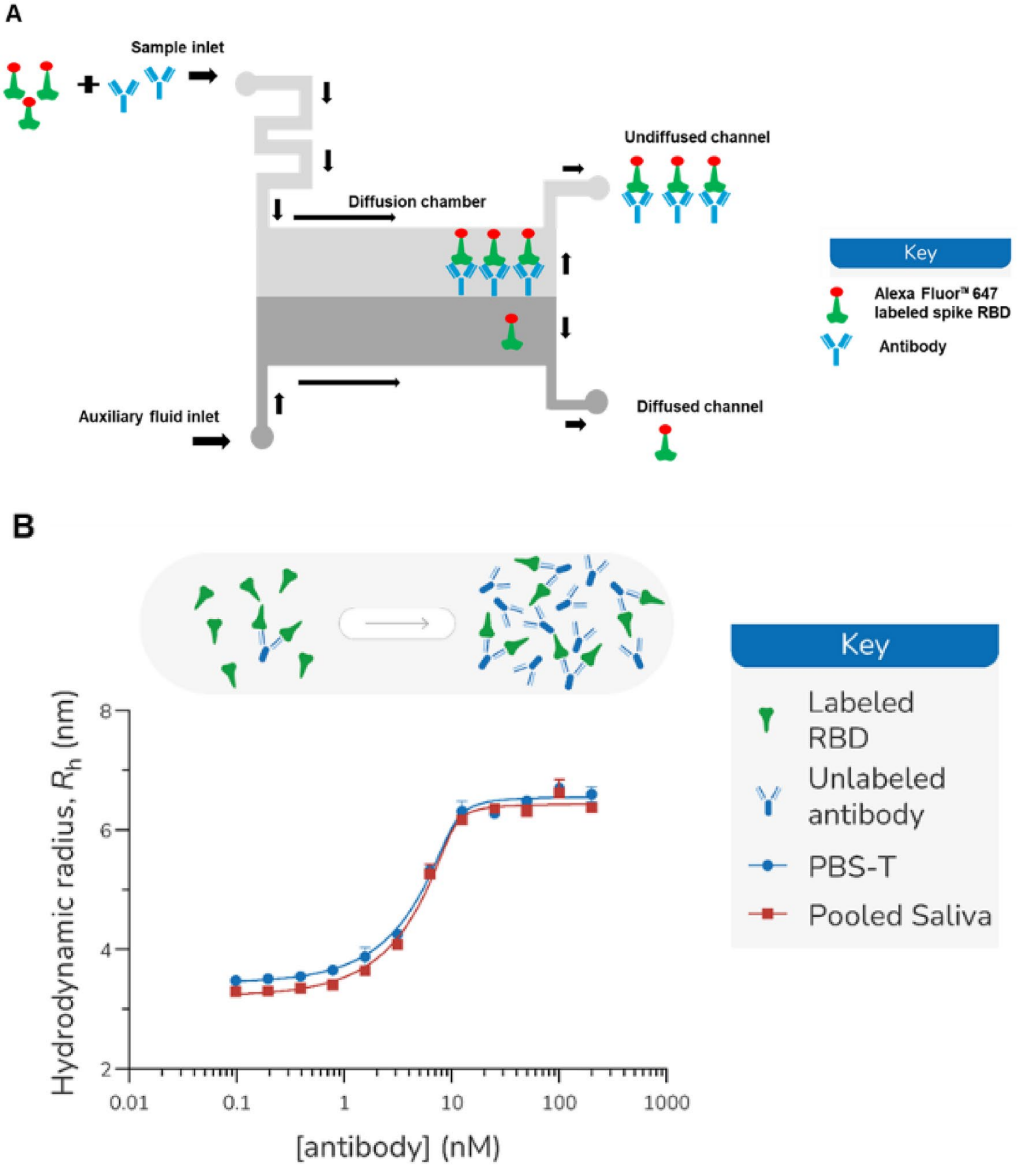
**A** Schematic diagram of the microfluidic chamber. Arrows indicate the direction of the liquid flow. The sample (analyte) and the auxiliary fluid are introduced into the microfluidic chamber through the inlets. The two streams of the liquid exploit laminar flow without convective mixing. The exchange of the molecules between the streams is through diffusion. Larger molecular complexes diffuse relatively slower than the smaller molecules. Therefore, the size (in hydrodynamic radius) of the molecule complexes can be measured through the ratio of the fluorescent intensity at the end of the chamber where the two stream splits. **B** Hydrodynamic radius for recombinant anti-spike monoclonal antibody and Alexa Fluor^TM^ 647 labeled spike RBD (10 nM) measured in PBS-T or 90% pooled saliva (supplemented with 10% PBS-T). Incubation of 10-nM RBD with 13 different titration points (one with no antibody and 12 geometrically spaced antibody concentrations) for 30 min prior to reading in duplicate.

Microfluidic Diffusional Sizing (MDS) provides insights into the conformation and stoichiometry of individual proteins and their complexes by measuring their absolute size (hydrodynamic radius, *R*_h_) directly in solution [17]. Significantly, the affinity of an interaction, *K*_D_, can be determined by monitoring the size of a protein against a titration of its binding partner. MDS measurement employs a microfluidic chamber system with defined dimensions, as illustrated in Fig.1A. Two inlets are designed for sample (analyte) input and auxiliary fluid input. The sample and the auxiliary fluid are drawn through the capillary effect to the diffusion chamber, where a diffusion gradient is formed across the two laminar flow fluids through an applied pressure. At the end of each diffusion chamber, the co-flow fluid is separated into two outlets and the fluorescence intensity of the labeled species collected in each outlet is recorded by a laser detector. The analyte size can be estimated by the ratio of the fluorescent intensity at each outlet channel [3]. As the measurement takes place entirely in solution, MDS has been successfully used to understand biologically relevant processes directly in biofluids, such as serum [16].

Here, we report the assessment of SARS-CoV-2 neutralization of saliva using MDS as a detection and characterization platform. We examined the feasibility of using MDS to detect neutralizing antibodies in saliva using fluorescently labeled ACE2. We qualitatively assessed the presence of neutralizing antibodies in patient saliva samples. We also demonstrated the feasibility of quantitative assessment of neutralizing antibody concentration and its affinity to the labeled spike trimer in patient saliva samples.

## Materials and Methods

### Pooled Saliva, Saliva Collected from Individual Donors, and Saliva Treatment

Pooled saliva collected pre-COVID was purchased from Lee Biosolutions (Item # 991-05-P-PreC-250). Saliva collection from individual donors post-COVID complied with the IRB protocol (IRB # Pro00100731). For Enzyme-linked Immunosorbent Assay (ELISA) and rVNT assays, the pooled saliva and saliva samples from individual donors were centrifuged at 2600×*g* for 15 min [8] and treated with 1% Triton X-100 for 1 h at room temperature. For MDS, saliva was first filtered through a 0.2-μm filter to remove cells. After cell removal, the saliva samples were used directly in the assay or treated with 1% Triton X-100 for 1 h.

### Recombinant Trimeric Spike Protein and the RBD Domain

Trimeric spike or the RBD domain was purchased from ExcellGene. Recombinant anti-spike antibody (PDB #7SBU, J08 Fab) was purchased from ExcellGene (SARS-CoV-2 Trimeric mAb-61 (XLG-SARSCoV2-mAb-3)), SN/Lot. 20511 [1, 45].

### ELISA

High-binding 96-well plates were coated with trimeric spike or RBD at 2 μg/ml and incubated at 4 °C overnight. The plate was blocked with 100-μL 3% milk at room temperature for 2 h and washed with 1×Phosphate-Buffered Saline supplemented with 0.1% Tween-20 (PBS-T). 25 μL of prediluted saliva sample (1:20 in 1×PBS) were added and incubated at room temperature for 2 h. The plate was washed with 1×PBS-T 3 times, and 50 μL of horseradish peroxidase (HRP)-conjugated anti-IgG (Invitrogen, 1:5000 diluted in 1×PBS-T with the supplement of 1% milk) were added and incubated at room temperature for 1 h. The plate was washed three times with 1×PBS-T and then 100 μl of 3,3’,5,5’-Tetramethylbenzidine (TMB) solution was added and incubated at room temperature. The reaction was stopped by 100 μL of 2 M sulfuric acid. The plate was scanned using a BioTek spectrometer at 450-nm wavelength.

### sVNT

The cPass SARS-CoV-2 Surrogate Virus Neutralization Test kit (Genscript) was used for neutralization verification. Saliva samples were 1:5 diluted with sample dilution buffer (supplied in the kit). Then, they incubated with HRP-labeled spike RBD with a volume ratio of 1:1. The mixture was incubated at 37 °C for 30 min and then added to the wells coated with hACE2 and incubated at 37 °C for 15 min. The wells were washed with the wash solution (supplied in the kit) 4 times. 100 μL of TMB solution was added and incubated at room temperature for 15 min. 50 μL of stop solution was added to stop the reaction. The plate was scanned using a BioTek spectrometer at 450-nm wavelength.

### Recombinant ACE2 and Spike Protein Labeling

ACE2 and spike RBD were each labeled overnight at 4 °C with Alexa Fluor™ 647 NHS ester (Thermo Fisher Scientific) at a dye-to-protein ratio of 3:1 and purified using a 1 mL Pierce® Desalting Column (Thermo Fisher Scientific) eluting with 1×PBS-T.

### Binding Model

The fluorescent fraction measured at the diffused and undiffused channels can be expressed as a function of the total protein and the ligand concentration [32]. The correlation between the diffusion rate and the molecular size of a protein in a polydisperse system had been measured [37, 48]. Therefore, the hydrodynamic radium *R*_h_ can be expressed as an equation of the weighted sum of the molecular mass of the unbound (diffused) and bound (undiffused) protein, measured from the fluorescent intensity of the two channels.

1 $$R_{h} = R_{h, free} + \left( {R_{h, complex} - R_{h, free} } \right)*\frac{{\left[ {PL} \right]}}{{n[P]_{tot} }},$$

where *R*_h_ is the hydrodynamic radius at equilibrium, *R*_h ,free_ is the hydrodynamic radius of the unbound protein, *R*_h ,complex_ is the hydrodynamic radius of the protein-ligand complex, $[PL]$ is the concentration of the bound protein and ligand, *n* is the number of binding sites of [P]_tot_, which was set at 2 in this study.

Using a second-order reaction protein-ligand binding model, we applied the thermal dynamics equation of the binding partners, the concentration of the binding partners and the ${K}_{D}$ is as follows:

2 $$n[P{]}_{tot}+[L{]}_{tot}\leftrightarrows \left[PL\right],$$

3 $$K_{D} = \frac{{\left( {n\left[ {P]_{tot} * } \right[L]_{tot} } \right) }}{{\left[ {PL} \right]}},$$

where *K*_D_ is the dissociation constant

4 $$n[P{]}_{tot}=n\left[P\right]+\left[PL\right],$$

5 $$[L{]}_{tot}=\left[L\right]+\left[PL\right].$$

From Eqs. 3, 4, and 5, we obtained a quadratic equation of $\left[PL\right]$.

6 $${[PL]}^{2}-\left[PL\right]*\left([L{]}_{tot}+n[P{]}_{tot}+{K}_{D} \right)+\left(n[P{]}_{tot}*[L{]}_{tot} \right)=0.$$

Therefore, $\left[PL\right]$ in the quadratic Eq. (6) can be solved as follows:

7 $$\left[PL\right]= \frac{({K}_{D}+[L{]}_{tot}+n[P{]}_{tot})-\sqrt{{(K}_{D}+[L{]}_{tot}+n[P{]}_{tot}{)}^{2}-4[L{]}_{tot} n[P{]}_{tot}}}{2}.$$

Substitute $\left[PL\right]$ in Eq. 1, we obtained Eq. 8, which expresses a function of ${R}_{h}$, the protein and ligand concentration, and the ${K}_{D}.$

8 $${R}_{h}={R}_{h, free}+\left({R}_{h, complex}-{R}_{h, free}\right)* \frac{({K}_{D}+[L{]}_{tot}+n[P{]}_{tot})-\sqrt{{(K}_{D}+[L{]}_{tot}+n[P{]}_{tot}{)}^{2}-4[L{]}_{tot} n[P{]}_{tot}}}{(2n[P{]}_{tot})}.$$

### Affinity Measurements of the Reference Monoclonal Antibody Binding to the Labeled Spike RBD

For antibody and spike RBD affinity measurements, two dilution series of monoclonal antibody were prepared to achieve a two-fold concentration series ranging from 400 to 0.195 nM using either PBS-T or pooled pre-COVID saliva (supplemented with 10% PBS-T) as the diluent. Antibody dilutions were mixed in a 1:1 ratio with a 20 nM solution of labeled SARS-CoV-2 spike RBD, prepared in the relevant assay matrix, to obtain a final RBD concentration of 10 nM, with a range of antibody concentrations from 200 to 0.098 nM. The binding curve was measured using both PBS-T and pooled saliva (90% saliva and 10% PBS-T) as the assay matrix. All samples were incubated for 30 min on ice prior to measurement [11]. The samples were loaded on the commercially available microfluidic chip (Fluidic analytics), and *R*_h_ was measured in duplicate using a Fluidity One-W (Fluidic Analytics) at the 1.5–8.0 nm size setting. The binding affinity, *K*_D_, was generated by non-linear least squares fitting to Eq. 8.

### Rapid Affinity-based Neutralizing Test (rANT)

To measure the ability of the neutralizing antibodies to disrupt the binding of the RBD or the SARS-CoV-2 spike trimer to ACE2, a rapid affinity-based neutralization test (rANT) was performed. To measure the *R*_h_ of unbound ACE2, a 5-nM solution of Alexa Fluor™ 647 labeled ACE2-Fc fusion was prepared in pooled saliva (free ACE2-Fc sample). For the ACE2/spike trimer complex sample, Alexa Fluor™ 647-labeled ACE2-Fc and SARS-CoV-2 spike trimer at concentrations of 5 nM and 10 nM were mixed with pooled saliva, respectively. To measure antibody-mediated inhibition, ACE2-Fc/spike trimer (5 nM/10 nM) was mixed with a neutralizing monoclonal antibody at a concentration of 180 nM in pooled pre-COVID saliva. Samples were incubated on ice for 1 h or 16 h prior to duplicate measurements on the Fluidity One-W serum using the commercially available microfluidic chips (Fluidic analytics), using the 1.5–8.0 nm size range setting.

### Quantitative Affinity-Based Neutralizing Test (qANT)

In the qANT assay, test samples of patient saliva were titrated against a pre-formed complex of ACE2-Fc and spike trimer. Samples were incubated for 16 h at 4 °C to maximize complex disruption, as for the rANT (see above). This titration was repeated for complexes formed from a constant ACE2 concentration of 5 nM but varying spike concentrations.

### Statistical Analysis

Statistical analysis was done using JMP Pro 17.1.0.

## Results

### Recombinant Anti-spike Monoclonal Antibody in Complex with Spike RBD Increases Its Hydrodynamic Radius

Figure 1A illustrates the sample loading and the fluidic flow of the MDS assay. Labeled spike RBD alone or pre-mixed with the recombinant antibody were loaded into the sample inlet. A co-flowing auxiliary fluid (water) is provided at a matched flow rate to that of the sample (0.2–1 µl/min depending on instrument size range setting). In the diffusion chamber, a diffusion gradient is formed across the two laminar fluid flows as the result of the Brownian motions. As previously determined, large-sized molecule complexes diffuse slower than the smaller-sized molecules [3]. If the spike RBD and the recombinant antibody form a large complex, the complex diffuses at a relatively slower rate than the spike RBD alone. Therefore, a hydrodynamic radius (*R*_h_) can be measured based on partitioning the fluorescently labeled ligand in the diffused and undiffused channel [28]. In this case, *R*_h_ is determined through the measured fluorescent signal intensity of the two outlets and calculated using Eq. 8.

Hydrodynamic radius (*R*_h_) measurements and other physical-chemical properties of the Alexa Fluor^TM^ 647 labeled spike RBD are listed in Table S1. Using the Alexa Fluor^TM^ 647-labeled spike RBD, we first compared the *R*_h_ of the Alexa Fluor-labeled spike RBD alone or in complex with the recombinant anti-spike monoclonal antibody in different matrices: PBS-T or saliva. For the *R*_h_ measurement of the complexes, labeled RBD protein (10 nM) was incubated with the reference neutralizing antibody (positive control 1000 nM) for 30 min on ice. There was no significant difference in the spike RBD *R*_h_ measured in PBS-T or pooled saliva (*R*_h_ = 3.31 ± 0.03 and 3.35 ± 0.06, respectively). In both matrices, the *R*_h_ of the spike RBD is significantly increased after it formed the complex with the recombinant antibody (*p* < 0.001). Spike RBD and antibody complexes formed irrespective of the matrix, whether PBS-T buffer or a more viscous saliva matrix (*R*_h_ = 6.54 ± 0.16 and 7.02 ± 0.01, respectively) (Table S2).

We further estimated the binding of spike RBD and the recombinant antibody. A two-fold titration down from the antibody concentration of 200 nM was tested in PBS-T and the pooled saliva (90% saliva and 10% PBS-T) (Fig. 1B). The *R*_h_ of the lowest four antibody concentrations was not significantly different from the *R*_h_ of the negative control. Under the experimental condition using a 10-nM spike RBD, MDS is sensitive to detect the spike RBD and the antibody complexes formed at 1.5-nM antibody concentration. The best-fit *K*_D_ values determined by non-linear least-square fitting (GraphPad Prism) were 340 pM (CI 95% 60, 750) for PBS-T and 250 pM (CI 95% 30, 560) for pooled saliva (Table 1). The overlapping confidence intervals indicate that the *K*_D_ values of the two binding partners are not statistically different. The inflection point estimated using best fitting was 1.64 (log concentration) (CI 95% 1.54, 1.75) for PBS-T and 1.6 (log concentration) (CI 95% 1.53, 1.69) for pooled saliva. The overlapping confidence intervals indicate that the inflection point of the curves was not significantly different. Therefore, the saliva matrix had no interference with the binding of the binding partners. Accordingly, all subsequent work in this study was performed using saliva as the assay matrix. All data presented in this study is an average of duplicate measurements.

**Table 1:** *K*_D_ of the interaction between Alexa Fluor^TM^ 647-labeled spike RBD and recombinant anti-spike monoclonal antibody in different matrices.

Matrix	*K*_D_ (pM)	95% CI (pM)
PBS-T	340	60–750
Pooled saliva (90%)	250	30–560

### Rapid Affinity-Based neutralization test (rANT) Detects Antibody Inhibition of the ACE2 and Spike RBD Complex Formation

**Fig. 2:**
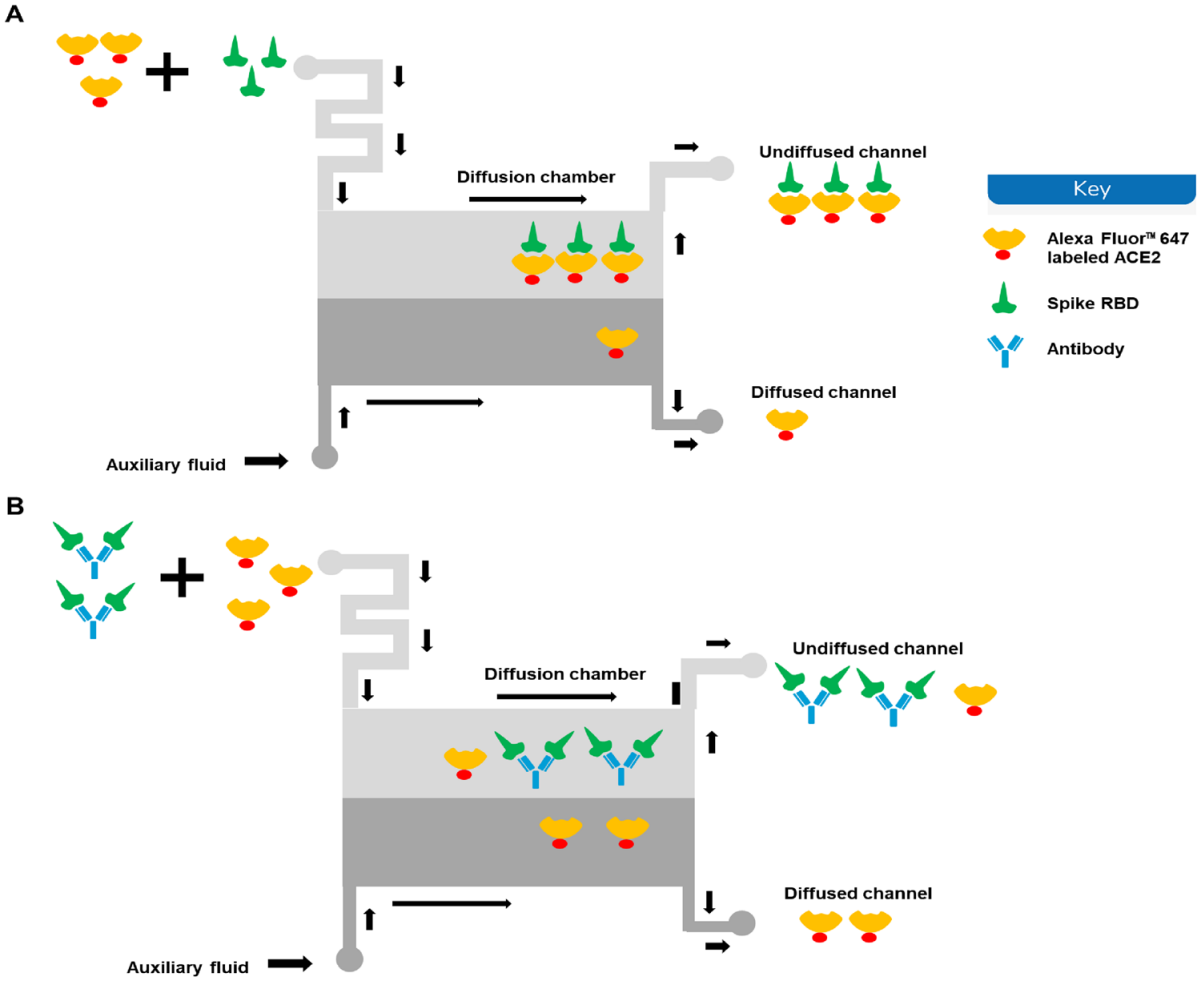
Schematic depict of the principles of the neutralizing antibody detection by microfluidic diffusional sizing. **A** When the labeled ACE2 is mixed with the spike trimer, the two binding partners form a complex that diffuses relative slower than the ACE2 alone. That results in an increase of the *R*_h_. **B** When the labeled ACE2 is mixed with spike trimer pre-incubated with the neutralizing antibody, the spike trimer can no longer bind to the labeled ACE2 as it is blocked by the antibody. Therefore, the measured *R*_h_ will be equivalent to the *R*_h_ of the labeled ACE2 alone.

**Fig. 3:**
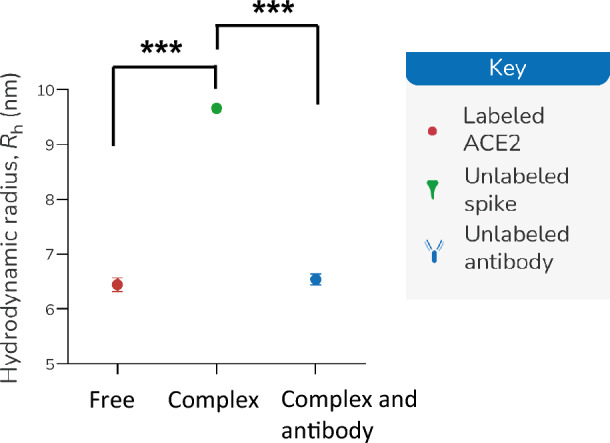
Affinity-based neutralization measurements (rANT) in pooled saliva. ACE2-Fc (5 nM) alone (blue symbol) and a mixture of 5-nM ACE2-Fc and 30-nM spike trimer (red symbol) were prepared in pooled saliva. Inhibition of ACE2-Fc/spike trimer incubated with 180-nM monoclonal antibody spiked into pooled saliva is shown in green. *** indicates a statistical difference (*p* < 0.001).

Once it is established that saliva did not affect the measurement of interactions, rANT was performed to verify that the disruption of a pre-formed complex by a neutralizing antibody could be measured. Samples of Alexa Fluor™ 647-labeled ACE2-Fc and the complexes of labeled ACE2-Fc and SARS-CoV-2 spike trimer were prepared using 90% pooled saliva (10% PBS-T) as the matrix. Additional complexes were prepared with a recombinant antibody spiked into the pooled saliva. The principle of MDS-based rANT test is illustrated in Fig. 2. When the labeled ACE2 is mixed with the spike trimer alone, a complex is formed between the ACE2 and the spike trimer, resulting in a measurable *R*_h_ signal reflecting the complex formation (Fig. 2A). When the labeled ACE2 is mixed with an equilibrated mixture of spike trimer and neutralizing antibody, the spike trimer can no longer bind to the labeled ACE2 as it is blocked by the antibody. Therefore, the *R*_h_ of the labeled ACE2 will remain unchanged (Fig. 2B). We previously determined the binding curve of the reference neutralizing antibody to the spike RBD titrated from 200 nM (Fig. 1B). Here, we used a reference antibody concentration of 180 nM to test the disruption of a pre-formed complex of labeled ACE2-Fc and spike trimer (Fig. 3). When the antibody is added, the antibody inhibits the formation of the complexes, indicated by the *R*_h_ change (free vs. complex and antibody *p* < 0.001; complex vs. complex and antibody *p* < 0.001). Therefore, it is feasible to use this rANT to assess the presence of neutralizing antibodies. To account for the fact that multivalent protein components were used for the rANT assays, the concentrations quoted refer to binding site concentrations rather than the protein-molecule concentrations, i.e., the ACE2-Fc dimer is assumed to have two binding sites, while the spike trimer is assumed to have three binding sites.

### rANT Detection of Patient Saliva Sample Virus Neutralization Capability

**Fig. 4:**
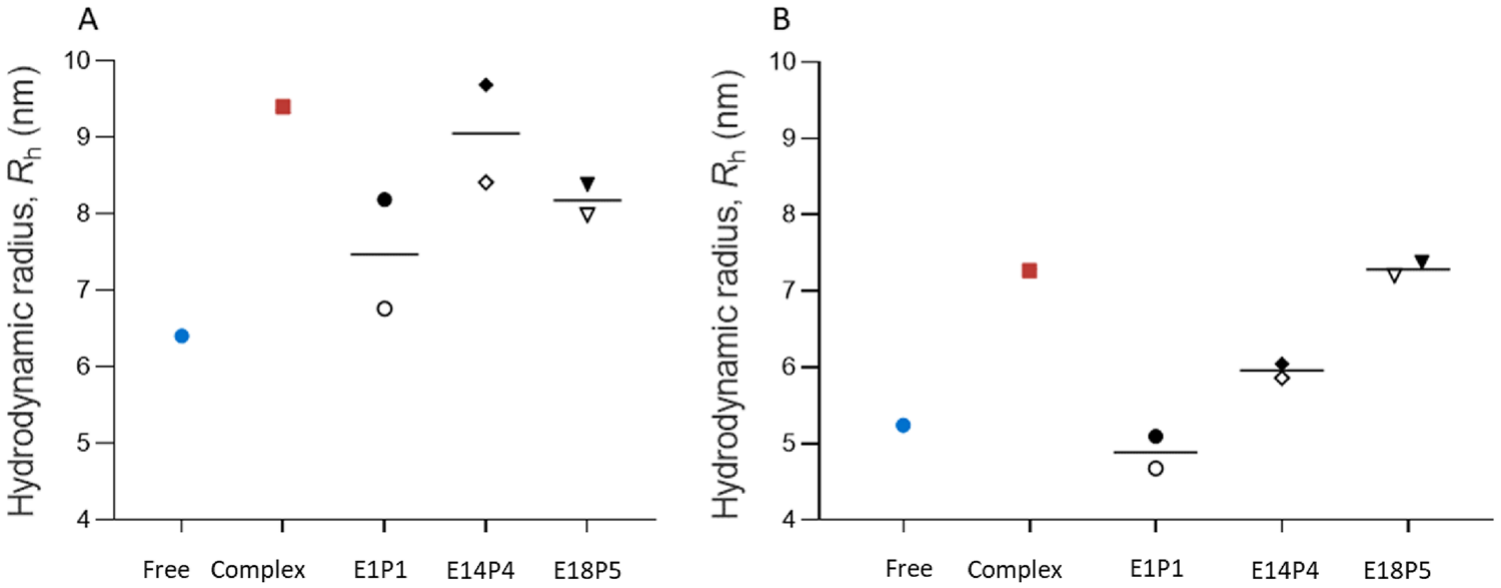
Qualitative measurements for neutralization in patient saliva (rANT). Samples were incubated for 1 **A** or 16 h **B** at 4 °C prior to measurement. Control measurements for ACE2 alone (blue symbols) and the ACE2 and spike trimer (red symbols) that were prepared in pooled saliva are shown. Measurements for patient saliva samples are indicated as E1P1, E14P4, and E18P5. The open symbols indicate the second measurement, which has approximately 1-h longer incubation time due to the time needed for taking each measurement sequentially.

The same experimental principle was used to analyze the patient saliva samples for the presence of neutralizing antibodies. We selected positive and negative spike RBD neutralization patient saliva to validate the rANT test. We estimated the total antibody titer of the saliva samples by ELISA (Table 2). The neutralization ability of the patient saliva samples was verified using cPass^TM^ sVNT (Genscript), using a cutoff limit of 30% inhibition according to the manufacture’s instruction (Table 3). For the rANT assay, the experiment was performed using a mixture comprised of 5-nM labeled ACE2 and 15-nM spike incubated in the presence of patient saliva on ice. We first measured the *R*_h_ after 1 h of incubation, Fig. 4A, black-filled symbols; repeat measurements were taken after the first-round measurement, which was approximately an hour later. Therefore, the second measurements have an estimated 1-h prolonged incubation time (Fig. 4A, black open symbols). The experiment was also repeated for 16-h incubation (Fig. 4B). Using three times of the instrument confidence interval (10%) as the inhibition cut off threshold, we calculated the *R*_h_ cutoff of positive and negative neutralizing from the following formula:

**Table 2:** Total antibody titer of the individual saliva sample determined by ELISA**.**

Sample	Spike RBD ELISA (ng/mL)	Spike Trimer (S1) ELISA (ng/mL)
E1P1	101.13 ± 12.26	96.7 ± 22.3
E14P4	289.04 ± 18.99	255.96 ± 76.27
E18P5	26.48 ± 3.1	28.4 ± 2.55

**Table 3:** Neutralization effect of the individual saliva sample determined by sVNT and rANT.

Sample	cPass^TM^ sVNT % signal inhibition RBD	cPass^TM^ sVNT qualitative result	MDS rANT qualitative result at 16 h incubation at 4 ºC
E1P1	44.34 ± 0.48	Positive	Positive
E14P4	36.92 ± 5.51	Positive	Positive
E18P5	20.45 ± 3.21	Negative	Negative

*R*_h_ (free) + [*R*_h_ (spike S1 and ACE2 complex) - *R*_h_ (free ACE2) ] * 30%

A saliva sample with a measured *R*_h_ >  = *R*_h_ (free) + [*R*_h_ (spike S1 and ACE2 complex) −*R*_h_ (free ACE2)] * 30% resulted in positive neutralizing. A saliva sample with a measured *R*_h_ < *R*_h_ (free) + [*R*_h_ (spike S1 and ACE2 complex) −*R*_h_ (free ACE2) ] * 30% resulted in negative neutralizing (Table 4). After 16-h incubation at 4 °C, the *R*_h_ measured for saliva samples (E1P1 and E14P4) are positive neutralizing, matching with the cPass^TM^ sVNAT qualitative analysis. We proceed with quantitative measurements using these two samples employing an overnight incubation time.

**Table 4:** Positive and negative neutralizing *R*_h_ cutoff.

Sample	Incubation hour	Mean *R*_h_ (nm) (n = 3)	Standard Deviation *R*_h_ (nm) (n = 3)	*R*_h free_ + 30% (*R*_h__complex_ –*R*_h free_)
Free ACE2	< = 2	6.43	0.09	7.37
In complex with Spike S1	< = 2	9.57	0.16	
Free ACE2	16	5.62	0.33	6.14
In complex with Spike S1	16	7.35	0.08	

### Quantitative Affinity-Based Neutralization Test (qANT) Reveals Binding Affinity and Binding Site Concentration in Saliva Sample

**Fig. 5:**
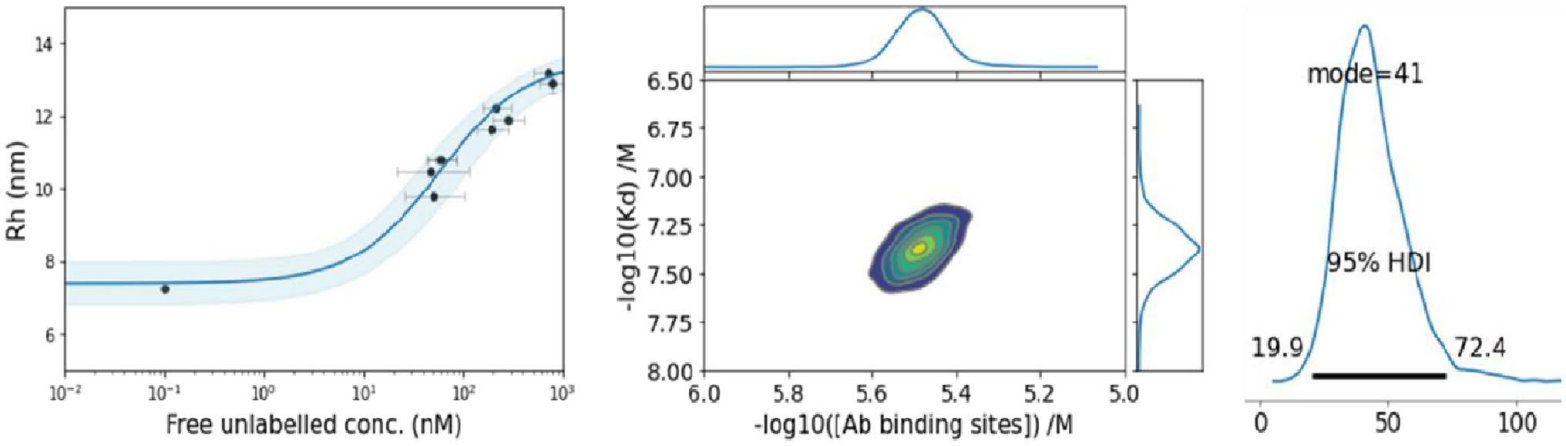
Bayesian *K*_D_ and binding site concentration assay analysis of ACE2 and spike protein. Indicated are the binding curve for the interaction (left panel), the probability density plot indicating the convergence of the *K*_D_ and spike binding site concentration based on the analysis (middle panel) and the modal distribution of the *K*_D_ derived from the fit of the data (right panel).

Having established that the E1P1 saliva was able to disrupt the binding of labeled ACE2-Fc and spike trimer, the concentration and affinity of these antibodies were determined using qANT. To process the ternary qANT data, it was necessary to determine the *K*_D_ of the binary interaction of ACE2 and spike trimer protein. The principle of determining the *K*_D_ of the binary interaction of ACE2 and spike trimer protein is illustrated in Fig. 2A. A *K*_D_ and binding site analysis based on Bayesian inference [41] was performed using pooled saliva at a concentration of up to 90% as the assay matrix (Fig. 5). A total of 18 steps of different combination of ACE2 and Spike concentration were tested. The range for the ACE2 used was from 0 to 90 nM. The spike percentage was used from 7 to 400%. A spike 100% solution was 3000 nM binding site. The *K*_D_ for the interaction was estimated by fitting Equation 8 at each step [27, 49]. We estimated the *K*_D_ for the ACE2 and the spike trimer is 41 nM (HDI 95% 19.9, 72.4). The binding site concentration was estimated to be 3.20 µM (HDI 95% 2.35, 4.36) (Table 5).

**Table 5:** Summary of the Bayesian *K*_D_ and binding site concentration analysis for the interaction of ACE2 and spike protein.

		***K***_**D**_** (nM)**	**[binding sites]** **(µM)**
[Spike protein] (3 µM)	Best-fit	41.0	3.20
	HDI (95%)	19.9-72.4	2.35 − 4.36

**Fig. 6:**
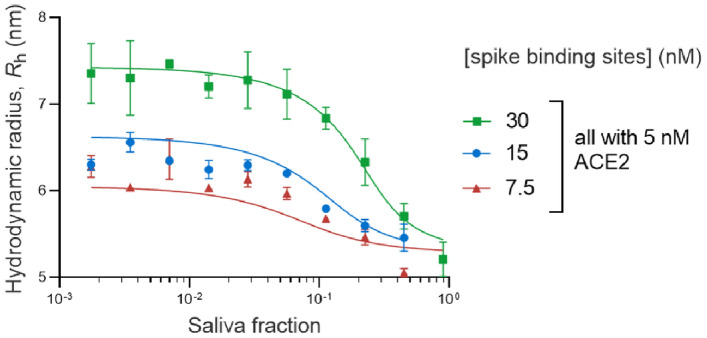
qANT analysis of E1P1 saliva. Three neutralization titration curves were measured for the disruption of a pre-formed complex of ACE2 (5 nM) and spike at 30 nM (green symbols), 15 nM (blue symbols), or 7.5 nM (red symbols).

Three titration curves were measured for the E1P1 saliva against mixtures consisting of 5-nM ACE2 and either 7.5-, 15-, or 30-nM spike trimer. The concentrations here refer to binding site concentrations rather than protein concentrations. The neutralization titration curves are shown in Fig. 6. In the presence of a higher fraction of saliva, there is more disruption of the complex formation and a corresponding decrease in the measured *R*_h_. The extent of the complex disruption is diminished when a higher concentration of spike protein is used. A summary of the qANT analysis for the anti-RBD antibodies in E1P1 saliva is shown in Table 6. E1P1 saliva has a *K*_D_ 3.2 nM (CI 95% 0.28, 38.95) and antibody binding site 120 nM (CI 95% 72.8, 521.9). The concentration here is given in units of binding sites, so if the binding molecule is secretory IgA (sIgA), then the concentration of immunoglobulin molecules will be ¼ of the value shown here. A qANT analysis for E14P4 saliva was also attempted (supplementary Fig. S1), but the data could not be analyzed to give a meaningful fit. This leads us to speculate that the neutralizing antibody in E14P4 saliva was too low in concentration to resolve the *K*_D_ and antibody binding site concentration against 5 nM ACE2 and 15-, 30-, or 60-nM spike trimer.

**Table 6.** Summary of the qANT analysis for saliva from saliva sample E1P1. Indicated are the best-fit values and 95% CI values from a least squares non-linear regression analysis.

*K*_D_ (nM)	95% CI (nM)	[Antibody binding site] (nM)	95% CI (nM)
3.20	0.28–38.95	120.0	72.8–521.9

## Discussion

### Direct Measurement of Binding Complex and Neutralization in Saliva

Antibody assays can be used to identify previous infections of SARS-CoV-2 with high sensitivity and specificity [39]. Compared to antigen screening, antibody screening resulted in six-fold more cases in children [19]. Although antibody assays against nucleocapsid and envelope have been developed with success, the anti-spike antibody test is broadly accepted as an assessment of immune response against SARS-CoV-2 [38]. An antibody meta-analysis study showed that spike antigen is more sensitive than nucleocapsid antigen [25]. Mucosal immune response is often underrated because secretory antibodies are compartmentalized and cannot be accurately evaluated using serological tests. In a child SARS-CoV-2 infection cohort, although IgA was detected in the saliva, there was no measurable IgA in the serum [23]. The sIgA in saliva is a polymeric antibody that is composed of an IgA dimer and the joining-chain (J chain) that binds to the secretory component, whereas IgA in serum exists as a monomer. The sIgA is structurally more stable than the monomeric IgA in serum [5]. sIgA in saliva remains detectable longer than the IgA in the serum [10]. IgA predominantly contributes to the early defense through neutralization of the spike protein; higher neutralization capacity correlates with better survival [10, 12]. Assessing mucosal antibodies, therefore, provides additional understanding of the adaptive immune response. Mucosal antibody detection may potentially lead to therapeutic innovations against viral pathogens.

The fluorescent labeling may affect the *R*_h_, diffusion rate, and *K*_D_ of the labeled protein [13, 18]. However, there is no consistent conclusion on how a protein binding affinity may be affected because proteins are different in size and charge. Alexa Fluor™ 647 labeling has been extensively used on ACE2 interaction with SARS-CoV-2 spike and functional studies of ACE2 [22, 29]. Therefore, we chose the Alexa Fluor™ 647-labeled ACE2 in the neutralization test. The affinity of Alexa Fluor™ 647-labeled ACE2 to the SARS-CoV-2 spike RBD is determined in this study (Table 5, Fig. 5). Label-free techniques such as Surface Plasmon Resonance (SPR) [4] and Biolayer Interferometry (BLI) [31] requires immobilization of one of the binding partners, rendering its application in the MDS-based testing.

Considering saliva is a biofluid with variable viscosity [14], we compared the binding of the labeled SARS-CoV-2 spike RBD and various concentrations of a recombinant antibody (PDB# 7SBU, J08 Fab, which is known for potent neutralization of spike RBD) in pooled saliva and PBS-T [45]. We found matching inflection points of the binding curve and equivalent *K*_D_ measurements in the buffer and saliva (Fig. 1B and Table 1). The changes in the viscosity in pooled saliva did not affect the binding property of the labeled spike RBD and the reference antibody.

The spike neutralization antibody (nAb) directly inhibits the binding of the spike protein and the ACE2 receptor through a neutralization mechanism that may involve divalent cross-linking of the neutralizing antibody and the spike protein [36]. The neutralizing ability has been commonly assessed using cell-based assay involving native virus, pseudotyped virus, or chimeric virus [2, 30]. A highly sensitive and specific alternative neutralization test using the surrogate virus has been developed (sensitivity: 98.3–100%, specificity: 85.7–100%) [44, 47]. However, none of these tests directly measure binding affinity. MDS, conversely, directly measures binding affinity. Using MDS measurement, the binding affinity of S1-ACE2 and serum nAb-S1 are 3.5–12.6 nM (95% CI) and 1.7–11.6 nM (95% CI), respectively [17]. MDS measurement also determined that in the sera matrix, the ACE2, S1, and sera nAb reach a ternary equilibrium, and it can be used to predict the neutralization potency [17].

We investigated the feasibility of using MDS-based assay to detect neutralizing antibodies in saliva samples. In the pooled saliva spiked with reference antibody, the virus spike trimer binding to ACE2 is clearly disrupted by the presence of the reference antibody (Fig. 3). The feasibility of MDS-based neutralization test on clinical saliva samples was also examined. After the preincubation of limited patient saliva samples with SARS-CoV-2 spike trimer, we were able to both qualitatively and quantitatively determine the inhibition of the salivary antibody to the spike – ACE2 complex. A total of five clinical samples were tested. The results of two neutralization-positive saliva (E1P1 and E14P4) and one negative saliva (E18P5) are consistent with the results determined by the FDA-approved cPass sVNT test (Table 3). The other two saliva samples (E6P2 and E10P3) were tested negative by the rANT assay. However, due to the sample volume being too low in E6P1 and E10P3, we were unable to verify the neutralization using the cPass sVNT test. Therefore, the results of E6P2 and E10P3 were not included. E18P5 has a very low amount of the total antibody titer (26.46 ± 3.1 ng/ml for spike RBD), presenting overall negative neutralization. The total antibody titer of E1P1 (101.13 ± 12.26 ng/ml for spike RBD) is only a third of that of E14P4 (289.04 ± 18.99 ng/ml). However, the neutralization capability of E1P1 is much stronger. It is not unusual for an individual to have a high total antibody response but a low neutralization because the neutralizing antibody is only a portion of the total antibody. Low virus-neutralizing antibodies, however, may correlate with high mortality [43]. This highlights the importance of the neutralization assay.

The concentration and affinity of the neutralizing antibody (assuming dominance of sIgA) were quantitatively measured. A total of 66 data points were collected for each qANT assay for each clinical sample. We demonstrate the feasibility of quantitative assay using a saliva sample and have determined the *K*_D_ and the concentration of neutralizing antibodies (Fig. 6, Table 6). Due to the continued mutation of SARS-CoV-2, the binding affinity and the cross-reactivity of antibodies against the variant spike protein are critical to be evaluated.

These results demonstrate the potential of MDS to measure the binding affinity of biomolecules in mixtures. This approach paves the way for the development of antibody tests to evaluate functional immunity in saliva. MDS-based assays are less laborious in test procedures compared to cell-based assays. Saliva sampling is less invasive and easier to be accepted by the public. In addition, MDS does not require heating samples for virus inactivation. Heat treatment of serum at 56 °C for 30 min causes an estimated 44% reduction of antibody activity in the serum samples [20]. For the rANT assay in this study, we filtered each saliva sample with a 0.2-μM filter, and the filtered samples were directly used for analysis. In the rVNT test, the incubation of the binding partner and the saliva was done at 4 ºC for 1 h or longer, the same conditions used for the serum rANT on SARS-CoV-2 antibody detection [15].

Due to the high variability of the methods used for antibody detection [34], a direct comparison of results from different studies and methods is nearly impossible. Evaluation of the commercially available assays for antibody titer has been conducted using the serum of the individuals infected with SARS-CoV-2 [35]. However, discrepancies in testing results remain when different methods were used to evaluate serum samples with low seroprevalence. The discrepancy is also shown when assessing the vaccine-elicited immune response [33]. Microfluidic Diffusional Sizing can be easily set up in all clinical conditions as it is easy to operate. Therefore, the results can be laterally compared among different studies.

The measurement of neutralizing antibodies using saliva as the matrix was shown to be reliably performed by MDS. There was measurable neutralization of the salivary antibody using a limited number of clinical saliva samples. The neutralization result is consistent with the result tested using the FDA-approved sVNT test. We show the feasibility of a quantitative affinity-based neutralization test (qANT) being developed to determine the concentration and affinity of the neutralizing antibody in saliva (assuming dominance of sIgA). Further optimization and clinical validation to determine sensitivity and accuracy using a large cohort of clinical samples is necessary.

### Supplementary Information

Below is the link to the electronic supplementary material.

Supplementary file1 (DOCX 53 kb)
