# Supplementary material for: Microfluidic Diffusional Sizing (MDS) Measurements of Secretory Neutralizing Antibody Affinity Against SARS-CoV-2
Source: Ann Biomed Eng. 2024 Mar 8;52(6):1653–64. doi: 10.1007/s10439-024-03478-0 (PMC11082020; doi:10.1007/s10439-024-03478-0)
Supplement: Supplementary file 1 — Supplementary file1 (DOCX 53 kb) [file 10439_2024_3478_MOESM1_ESM.docx]

**Supplementary materials:**

**Table S1.** Yield and hydrodynamic radius of the Alexa FluorTM 647 labeled RBD stock solution used for the assay development and affinity determination studies.

|  | **Pooled RBD stock solution** |
| --- | --- |
| **Protein mass concentration (mg/mL)** | 0.23 |
| **Protein molar concentration (µM)** | 4.54 |
| **Alexa Fluor™ 647 concentration (µM)** | 13.92 |
| **Labeling ratio (dye:protein)** | 3.1 |
| **Protein amount (µg)** | 18 |
| **Sample volume (µL)** | 80 |
| **Molecular weight (kDa)** | 50 |
| ***Predicted hydrodynamic radius (*R*_h_) (nm)** | 3.18 nm |
| **Observed hydrodynamic radius (*R*_h_) (nm)** | 3.3 nm |
| ***Folded predicted molecular weight (kDa)** | 56 |

**Table S2. H**ydrodynamic radius of the Alexa FluorTM 647 labeled RBD (free form) and the complex formed with the control antibody in PBST or pooled saliva matrix.

| Matrix | Spike RBD  *R*_h_ (free) | Spike RBD and Antibody complex  *R*_h_ (complex) |
| --- | --- | --- |
| PBS-T | 3.31 ± 0.03 | 6.54 ± 0.16 |
| Pooled saliva (90%) | 3.35 ± 0.06 | 7.02 ± 0.01 |

**Figure S1.**

**
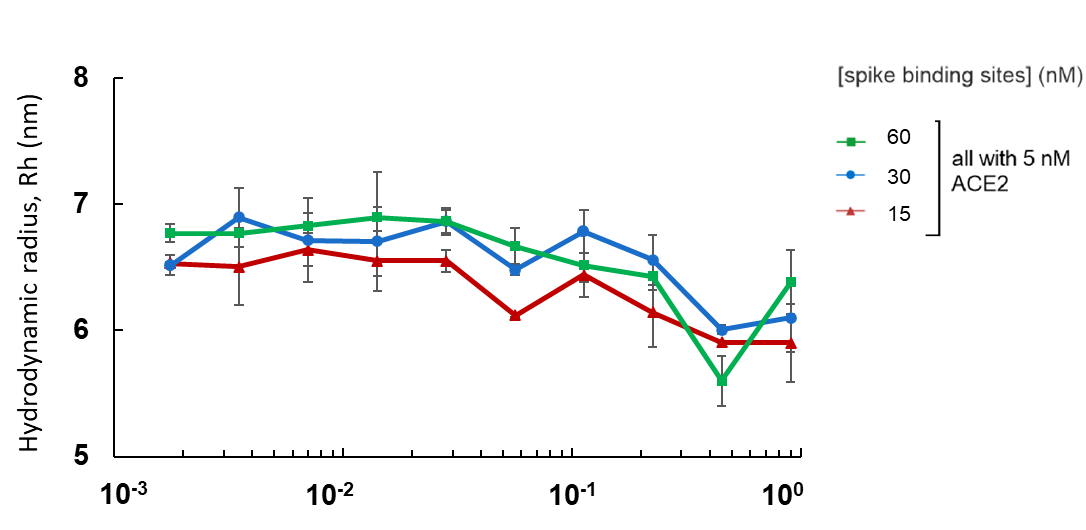
**

**Figure S1:** qANT analysis of E14P4 saliva. Three neutralization titration curves were measured for the disruption of a preformed complex of ACE2 (5 nM) and spike at 15 nM (red symbols), 30 nM (blue symbols) or 60 nM (green symbols).
